# The Electronic Structure and Optical Properties of Anatase TiO_2_ with Rare Earth Metal Dopants from First-Principles Calculations

**DOI:** 10.3390/ma11020179

**Published:** 2018-01-24

**Authors:** Kefeng Xie, Qiangqiang Jia, Yizhe Wang, Wenxue Zhang, Jingcheng Xu

**Affiliations:** 1 State Key Laboratory of Plateau Ecology and Agriculture, Qinghai University, Xining 810016, China; 2015990037@qhu.edu.cn (Q.J.); 2014990051@qhu.edu.cn (Y.W.); 2 Lanzhou Petrochemical Research Center, Petrochina, Lanzhou 730060, China; 3 School of Materials Science and Engineering, University of Shanghai for Science and Technology, Shanghai 200093, China; jchxu@usst.edu.cn; 4 Shanghai Innovation Institute for Materials, Shanghai 200444, China

**Keywords:** rare earth metal atoms, anatase TiO_2_, electronic and optical properties

## Abstract

The electronic and optical properties of the rare earth metal atom-doped anatase TiO_2_ have been investigated systematically via density functional theory calculations. The results show that TiO_2_ doped by Ce or Pr is the optimal choice because of its small band gap and strong optical absorption. Rare earth metal atom doping induces several impurity states that tune the location of valence and conduction bands and an obvious lattice distortion that should reduce the probability of electron–hole recombination. This effect of band change originates from the 4*f* electrons of the rare earth metal atoms, which leads to an improved visible light absorption. This finding indicates that the electronic structure of anatase TiO_2_ is tuned by the introduction of impurity atoms.

## 1. Introduction

Photocatalytic reactions on the TiO_2_ surface is one of the most active areas of research in photochemistry [1]. Anatase TiO_2_ is the best photocatalytic material due to its high abundance, low price, excellent chemical stability, and indirect band gap [2,3,4]. Therefore, TiO_2_ is an important photocatalyst in organic synthesis [5] in addition to its applications in solar cells [6,7,8], photocatalytic water splitting [9,10,11], and organic pollution degradation [12,13,14]. However, the wide band gap of TiO_2_ limits its uses to only the ultraviolet range, thereby resulting in the waste of a high amount of solar energy resources [15]. The TiO_2_ band gap can be engineered by creating lattice defects, such as vacancies, substitution, and interstitial space, which in turn change its electrical, thermal, magnetic, and optical properties [16,17,18]. Moreover, the photoexcited electron-hole pairs tend to recombine easily, which leads to low photoquantum efficiency. Researchers have utilized many effective methods to address these issues and have achieved few and partially satisfactory results in such fields as noble metal loading [19,20], dye-sensitizing [21,22], semiconductor mixing [23,24], and ion doping [25,26].

Ion doping using metals or nonmetals is one of the most effective modification methods and has attracted extensive attention from scientific researchers. Metal ions are generally incorporated into the lattice by occupying the lattice Ti sites, such as Fe- [25,26], Zn- [16,27], and La-doping [19]. An additional limiting factor for photocatalytic applications is the relatively short life of the excited electron-hole pairs in TiO_2_. Moreover, impurity atoms can create new defective complexes and introduce deep-defect levels that may act as trapping centers, thereby favoring charge separation [28]. Lanthanide is one of the rare earth metal impurities used to dope anatase TiO_2_ because it can improve the absorption in the visible light region [19]. Incorporation of lanthanide into anatase TiO_2_ leads to the appearance of La 4*f* states near the top of the valence band, whereas substitutional La doping is also predicted to shift the optical absorption spectra to the long wavelength region. La-doped anatase nanotube characterization shows band gap narrowing in the doped nanostructures and increases the photocatalytic activity for the system [19].

In this work, we investigated the impact of rare earth metal doping on the structural, electronic, and optical properties of anatase TiO_2_ based on first-principles calculations. We calculated the defect formation energy, band structure, density of states (DOS), and absorption spectrum of anatase TiO_2_ doped by rare earth metal atoms. The incorporation of rare earth metals into anatase TiO_2_ can increase the photocatalytic activity in the visible light region regardless of the dopant atoms.

## 2. Method and Calculation

**Figure 1:**
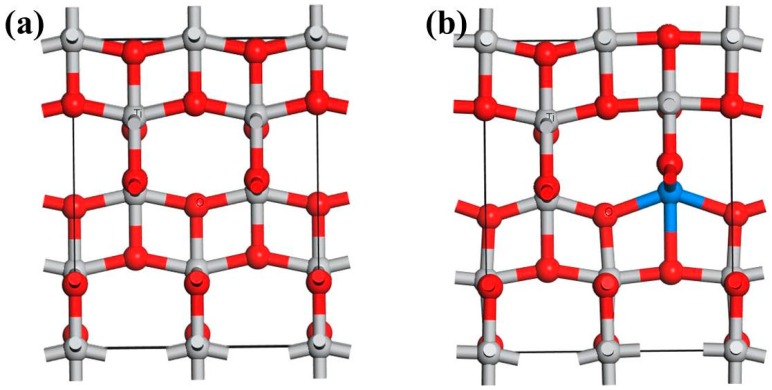
A 3-D model of anatase TiO_2_ (**a**) and with rare earth metal dopant (**b**); blue atom is a rare earth metal atom.

A 3-D model of the crystallographic structure of anatase TiO_2_ was built conforming to the standard database in Material Studio, showing that the crystal is tetragonal (space group D4H-19 with a = b = 3.776 Å and c = 9.486 Å). A 2 × 1 × 1 (7.552 × 3.776 × 9.486 Å, 17TiO_2_) supercell was used in this study (Figure 1a). The model of anatase TiO_2_ doped by a rare earth metal atom is shown in Figure 1b. The calculations were performed using density functional theory (DFT), as implemented in the Dmol^3^ software in the Materials Studio package [29,30]. Generalized gradient approximation was used with the Perdew–Burke–Ernzerhof exchange correlation function [31]. The localized double numerical basis sets with polarization functions were used to expand the Kohn–Sham orbitals, and the periodic boundary condition was used to simulate infinite TiO_2_. The cut-off energy for plane-wave expansion was set to 400 eV. The Brillouin zone was sampled using a 6 × 6 × 1 Gamma-centered Monkhorst–Pack grid [32]. The energy convergent criterion was 10^−4^ eV per atom during structural relaxation. The forces on all relaxed atoms were less than 0.02 Ha/Å. During the geometry optimization, the whole of the system was relaxed. For the DOS calculation, the k-point was set to 9 × 9 × 1 to achieve high accuracy. Test calculations using a larger supercell of 2 × 2 × 1 (7.552 × 7.552 × 9.486 Å, 28TiO_2_) and a higher cut-off energy of 500 eV were performed, which showed less than 3% improvement to the simulation accuracy. Therefore, the methodology we use is representative.

The co-doped system stability was estimated using the defect formation energy $E_{f}$. $E_{f}$ is defined as follows:

$$E_{f}=E_{d}-E_{p}+\mu_{Ti}-\mu_{Re}$$

where $E_{d}$ is the DFT total energy of the doped supercell, $E_{p}$ is the energy of the supercell without impurities, and $\mu_{Re}$ and $\mu_{Ti}$ are the total energies of the bulk rare earth metals and *Ti* metals per atom, respectively.

## 3. Results and Discussions

### 3.1. Defect Configurations and Formation Energy

After structure optimization of the pure anatase TiO_2_, the lattice parameters were a = b = 3.776 Å and c = 9.486 Å, which all agreed with current experimental (a = b = 3.7971 Å, c = 9.5790 Å) [33] and theoretical results [20,27]. Given the different atomic radius between dopants and host lattice atoms, the crystal structures will change differently in different doping models. The bond lengths and defect formation energies for substitutional rare earth metal doping configurations are shown in Table 1. The Ti–O bond length of the pure anatase TiO_2_ supercell is 1.930 Å, which is significantly different from that of the rare earth metal-doped TiO_2_. The rare earth elements include the lanthanide elements, namely, scandium and yttrium. The lanthanide elements were used as dopant atoms in this study. The atomic radii of the lanthanide elements were more than that of the Ti atom; thus, the Ti–O bond length was shortened, and the O–re (re stands for the rare earth metal atom) bond length was elongated. From the global aspect, the O–re bond length was decreased in increments of the lanthanide element atomic number. This special phenomenon originates from the lanthanide contraction, in which the atomic radius of the lanthanide elements significantly decreases while the atomic number increases.

To understand the relative doping difficulty under different growth conditions, we calculated the doping formation energy of different doping systems. The formation energies of the different doped systems under two extreme conditions were obtained, and the results are summarized in Table 1. As shown in Table 1, the formation energies of all models are positive, which indicated an endothermic process. In addition, the energies of the systems increased. Subsequently, the system stability decreased when anatase TiO_2_ was doped by rare earth metals. Moreover, the formation energy of the dopant systems initially increased and then decreased in increments of the lanthanide element atomic number. When Nd, Pm, Sm, Eu, Gd, Tb, Dy, Ho, and Er atoms were used to dope anatase TiO_2_, the formation energies exceeded 10 eV (equivalent to 998 KJ/mol); hence, the systems should be difficult to form. We only choose elements with formation energy less than 10 eV for further investigation. Thus, the systems of La, Ce, Pr, Tm, and Yb-doped anatase TiO_2_ were selected.

### 3.2. Electronic Structure and Properties

**Figure 2:**
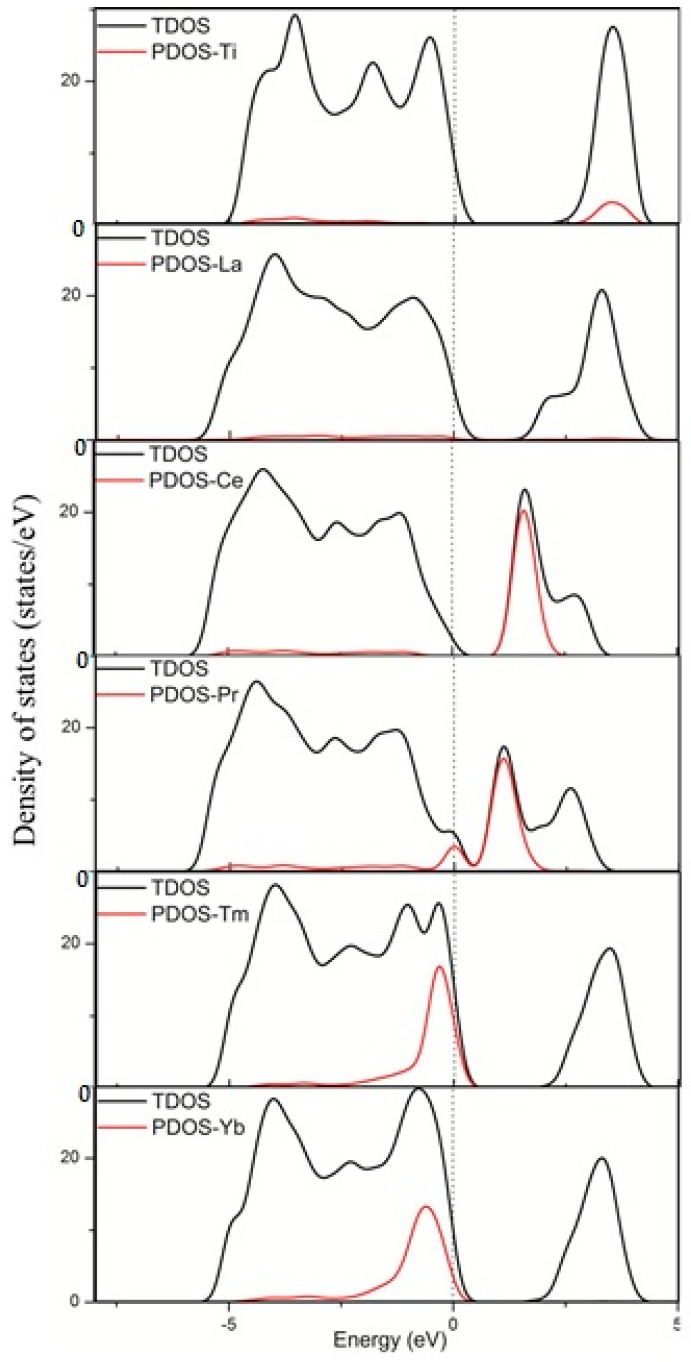
Calculated total DOS and PDOS of the pure TiO_2_ and TiO_2_ doped by La, Ce, Pr, Tm, and Yb. The Fermi levels are set at the zero of energy.

The incorporation of foreign ions into the host lattice does not only cause structural distortions but also modifies the electronic structures of TiO_2_, such as the shifts of valence and conduction bands edges and the introduction of impurity states in the band gap. Changes in the electronic properties have important effects on the TiO_2_ photocatalytic performances. Different doping models have different influences on the electronic structures by way of introducing different types of impurity states [34,35,36,37,38]. The position of the impurity states in the gap is important for regulating the electronic properties and photocatalytic activity. When the impurity states are near the valence or conduction bands, they are shallow impurity states. Shallow impurity levels are favorable to the separation of photoexcited electron-hole pairs because they can trap the photoexcited electrons and holes, thereby inhibiting the recombination of photoexcited carriers. In addition, the defect sites induced by the dopants can act as trap centers of the photoexcited electrons and holes, thereby further promoting carrier separation. However, the impurity states near the middle of the band gap are deep impurity states. These deep impurity states can easily become the recombination centers of the photoexcited electron-hole pairs, especially at high doping concentration structures. In this study, we present different rare earth metal atom doping configurations. On the basis of the discussions above, we plotted the DOS and PDOS for La, Ce, Pr, Tm, and Yb doping systems, as shown in Figure 2. For comparison, the DOS and PDOS for pure anatase TiO_2_ are included.

**Figure 3:**
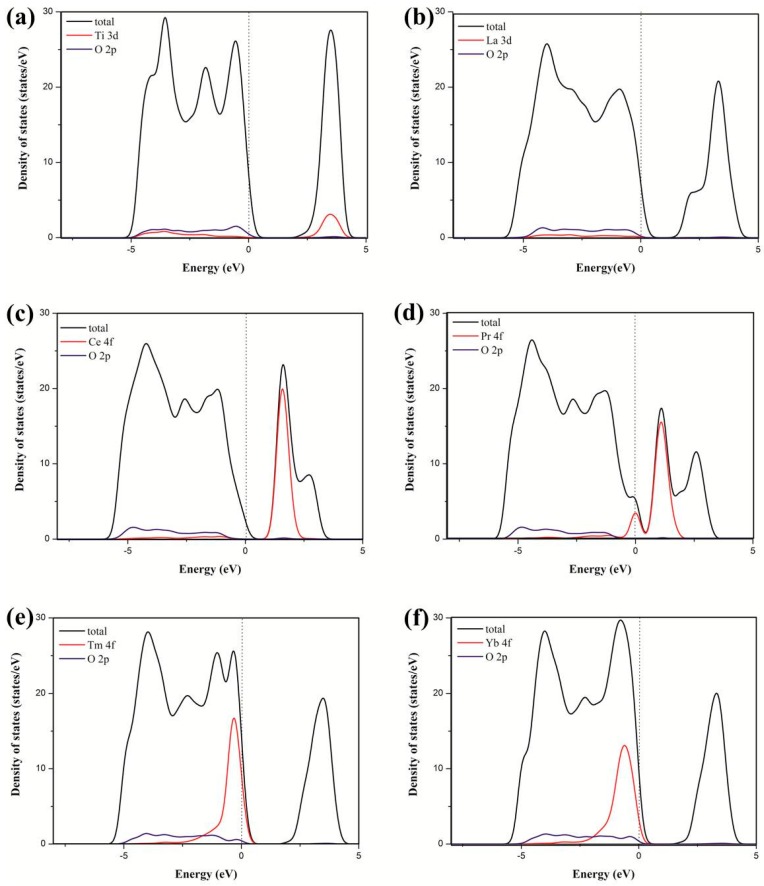
Calculated local partial DOS of the TiO_2_ (**a**) and TiO_2_ doped by La (**b**), Ce (**c**), Pr (**d**), Tm (**e**), and Yb (**f**). The Fermi levels are set at the zero of energy.

Figure 2 shows that the incorporation of rare earth metal atoms into the anatase TiO_2_ lattice causes shifts of the valence and conduction band edges and the introduction of impurity states in the band gap. Different rare earth metal atoms were introduced to the anatase TiO_2_ lattice, showing significant differences in the valence and conduction bands. The Ti states mainly contributed to the conduction bands in pure TiO_2_, whereas the Ce states contributed to the conduction bands in the dopant system. Moreover, the Pr states contributed to the valence and contribution bands in the dopant system, and the Tm and Yb states contributed to the valence bands in the dopant system. However, the La states rarely contributed to the valence and contribution bands in the dopant system. Figure 3 shows that the 4*f* orbital of the rare earth metal atom tunes the location of the valence and conduction bands. For pure TiO_2_, the valence bands mainly consist of the O 2*p* states, as well as a small contribution from the Ti 3*d* states. The conduction bands mainly consist of the Ti 3*d* states, with a small contribution from the O 2*p* states. For TiO_2_ doped by La, the La 3*d* states rarely contribute to the valence and contribution bands in the dopant system. For TiO_2_ by Ce, the Ce 4*f* states contribute to the conduction bands. For TiO_2_ by Pr, the Pr 4*f* states contribute to the valence and contribution bands. For TiO_2_ by Tm or Yb, the 4*f* states of the two atoms contribute to the valence bands. Therefore, these results are consistent with the PDOS in Figure 2. This finding indicated that the electronic structure of anatase TiO_2_ is tuned by the introduction of impurity atoms.

### 3.3. Optical Properties

**Figure 4:**
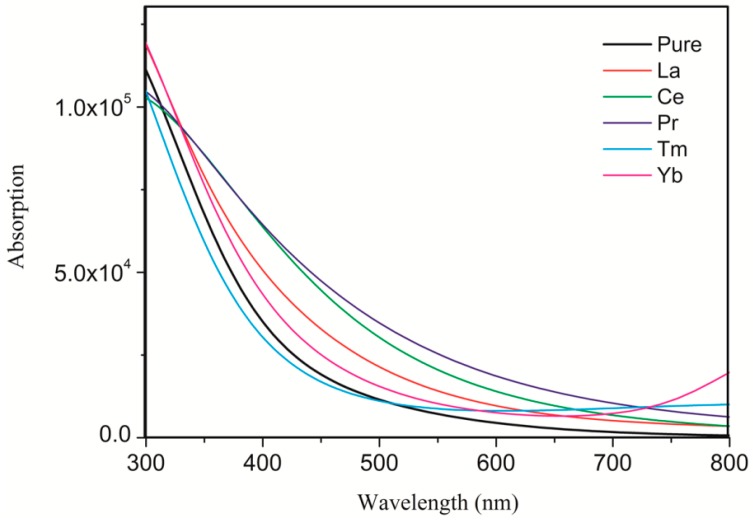
Calculated UV–visible absorption spectra of the pure and differently doped systems.

**Figure 5:**
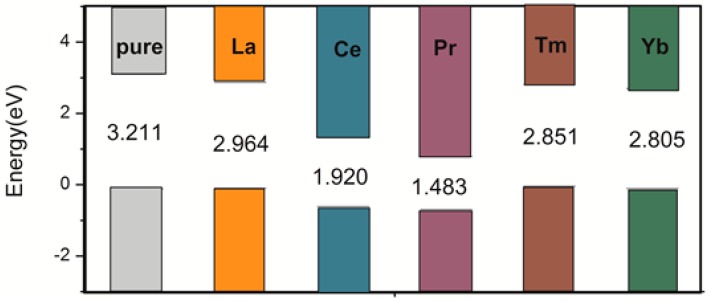
Change in the conduction band minimum and valence band maximum of the anatase TiO_2_ doped by single La, Ce, Pr, Tm, and Yb atoms. The Fermi levels are set at the zero of energy.

To better demonstrate the improvement in photocatalytic activity, we calculated the UV–visible optical absorption spectra for pure and doped systems, as plotted in Figure 4. The pure anatase TiO_2_ can only absorb UV light because of the wide band gap. After the rare earth metal atom doping, due to the significant changes in electronic structures by changing the composition of the energy bands and introducing the impurity states in the band gap, the solar absorption edge would be extended toward the visible light region from the UV light region, leading to the red-shift of the absorption edge. The absorption spectra were correlated with the band gap of the dopant systems. The band gap was narrower, and the absorption spectra were more red-shifted. As shown in Figure 5, the calculated band gap of pure TiO_2_ is 3.211 eV, which is in agreement with the experimental band gap value of 3.23 eV. The La-, Ce-, Pr-, Tm-, and Yb-doping TiO_2_ band gaps are 2.964, 1.920, 1.483, 2.851, and 2.805 eV, respectively, which are consistent with the tendency of the absorption spectra. In comparison, Ce- and Pr-doping can not only extend the light absorption spectra toward the visible light region but can also significantly enhance the visible light absorption. Moreover, Ce- and Pr-doped anatase TiO_2_ were more stable because of the low formation energy. Therefore, Ce- and Pr-doping are ideal to effectively utilize solar energy and improve the TiO_2_ photocatalytic activity in the visible light region.

## 4. Conclusions

We performed DFT calculations to study the modification mechanism of rare earth metal atom doping with respect to anatase TiO_2_ electronic structures. The calculated results indicate that the doping effects lead to remarkable structural distortions, which cause the local internal electric field in the lattice. This phenomenon changes the dipole moments and greatly promotes the separation of photoexcited electron-hole pairs. The doping effects change the electronic structures by introducing the impurity states in the band gap due to the hybridization between the host and dopants. Therefore, the doping influence on the electronic structures is significantly localized. This phenomenon does not only result in great band gap narrowing, which promotes visible light absorption, but also greatly inhibits the recombination of photoexcited electron-hole pairs. Thus, this result significantly leads to improvement in TiO_2_ photocatalytic activity in the visible light region.

## Figures and Tables

**Table 1:** Calculated bond length (Å) and defect formation energies (eV) of pure and doped TiO_2_.

Model	Electronic Configuration	d_Ti-O_	d_re-O_	$E_{f}$
Pure		1.930	-	-
La	[Xe]5d^1^6s^2^	1.835	2.180	9.34
Ce	[Xe]4f^1^5d^1^6s^2^	1.846	2.138	5.16
Pr	[Xe]4f^3^6s^2^	1.847	2.132	5.36
Nd	[Xe]4f^4^6s^2^	1.844	2.125	14.64
Pm	[Xe]4f^5^6s^2^	1.845	2.120	18.14
Sm	[Xe]4f^6^6s^2^	1.841	2.125	23.01
Eu	[Xe]4f^7^6s^2^	1.829	2.142	28.84
Gd	[Xe]4f^7^5d^1^6s^2^	1.848	2.100	28.70
Tb	[Xe]4f^9^6s^2^	1.850	2.103	20.96
Dy	[Xe]4f^10^6s^2^	1.844	2.106	16.20
Ho	[Xe]4f^11^6s^2^	1.845	2.101	11.26
Er	[Xe]4f^12^6s^2^	1.846	2.097	10.81
Tm	[Xe]4f^13^6s^2^	1.848	2.092	8.08
Yb	[Xe]4f^14^6s^2^	1.849	2.082	7.95
